# Next generation metronomic chemotherapy—report from the Fifth Biennial International Metronomic and Anti-angiogenic Therapy Meeting, 6–8 May 2016, Mumbai

**DOI:** 10.3332/ecancer.2016.689

**Published:** 2016-11-02

**Authors:** Pan Pantziarka, Lisa Hutchinson, Nicolas André, Sébastien Benzekry, Francesco Bertolini, Atanu Bhattacharjee, Shubhada Chiplunkar, Dan G. Duda, Vikram Gota, Sudeep Gupta, Amit Joshi, Sadhana Kannan, Robert Kerbel, Mark Kieran, Antonella Palazzo, Aparna Parikh, Eddy Pasquier, Vijay Patil, Kumar Prabhash, Yuval Shaked, Giselle Saulnier Sholler, Jaroslav Sterba, David J. Waxman, Shripad Banavali

**Affiliations:** 1 Anticancer Fund, Brussels, 1853 Strombeek-Bever, Belgium; 2 The George Pantziarka TP53 Trust, London, UK; 3 Nature Reviews Clinical Oncology, London N1 9XW, UK; 4 Service d’hématologie et Oncologie Pédiatrique, Centre Hospitalo-Universitaire Timone Enfants, AP-HM, Aix-Marseille Université, INSERM, CRO2 UMR_S 911, Marseille, France; 5 Metronomics Global Health Initiative, Marseille, France; 6 Inria team MONC and Institut de Mathématiques de Bordeaux, Talence, France; 7 European Institute of Oncology, via Ripamonti 435, 20141 Milan, Italy; 8 Malabar Cancer Centre, Thalassery, India; 9 ACTREC, Tata Memorial Centre, Kharghar, Navi Mumbai 410210, India; 10 Department of Radiation Oncology, Massachusetts General Hospital, Boston, MA, USA; 11 Tata Memorial Hospital, Mumbai, India; 12 Biological Sciences Platform, Sunnybrook Research Institute, University of Toronto, Toronto, ON, Canada; 13 Dana-Farber Cancer Institute, Boston, MA, USA; 14 PRA Health Sciences, Mumbai, India; 15 INSERM UMR 911, Centre de Recherche en Oncologie Biologique et Oncopharmacologie, Aix-Marseille University, Marseille, France; 16 Department of Cell Biology and Cancer Science, Rappaport Faculty of Medicine, Technion-Israel Institute of Technology, Haifa, Israel; 17 Helen DeVos Children’s Hospital, Grand Rapids, Michigan, USA; 18 Department of Pediatric Oncology, University Hospital Brno and Faculty of Medicine, Masaryk University, Cernopolni 9, 613 00 Brno, Czech Republic; 19 International Clinical Research Center, St. Anne’s University Hospital and RECAMO, Masaryk Memorial Cancer Institute, Brno, Czech Republic; 20 Department of Biology, Boston University, Boston, MA 02215, USA; 21 Division of Medical Senology, European Institute of Oncology, Via Ripamonti 435, 20141, Milan, Italy

**Keywords:** Metronomic chemotherapy, drug repurposing, anti-angiogenics, cancer, LMIC

## Abstract

The 5^th^ Biennial Metronomic and Anti-angiogenic Therapy Meeting was held on 6^th^ – 8^th^ May in the Indian city of Mumbai. The meeting brought together a wide range of clinicians and researchers interested in metronomic chemotherapy, anti-angiogenics, drug repurposing and combinations thereof. Clinical experiences, including many from India, were reported and discussed in three symposia covering breast cancer, head and neck cancers and paediatrics. On the pre-clinical side research into putative mechanisms of action, and the interactions between low dose metronomic chemotherapy and angiogenesis and immune responses, were discussed in a number of presentations. Drug repurposing was discussed both in terms of clinical results, particularly with respect to angiosarcoma and high-risk neuroblastoma, and in pre-clinical settings, particularly the potential for peri-operative interventions. However, it was clear that there remain a number of key areas of challenge, particularly in terms of definitions, perceptions in the wider oncological community, mechanisms of action and predictive biomarkers. While the potential for metronomics and drug repurposing in low and middle income countries remains a key theme, it is clear that there is also considerable potential for clinically relevant improvements in patient outcomes even in high income economies.

## Introduction

The meeting was organised such that the first day (Friday 6^th^ May) was devoted to an extended workshop on clinical trial design and translational research for metronomic studies, whereas days two and three included a series of plenary talks, symposia on specific disease areas and other presentations. The formal inauguration of the meeting, by Professor Shripad Banavali (Tata Memorial Hospital, India), took place on the morning of Saturday 7th May. The following detailed report of the meeting uses a thematic structure rather than a strictly chronological one.

## Trial Design

### Why metronomics?

Vikram Gota, (Tata Memorial Centre/ACTREC, India) discussed the pharmacokinetics (PK) of low dose chemotherapy. The starting point is that metronomic chemotherapy (MC) may be defined as a ‘frequent, regular administration of drug doses designed **to maintain low, but active**, range of concentrations of chemotherapeutic drugs during prolonged periods of time without inducing excessive toxicities’ [1]. Despite more than a decade of clinical studies, very little work has been published on the PK data related to low-dose chemotherapy, primarily due to an underestimation of the importance of plasma/tumour concentrations of drugs and their biological effects. This is particularly the case when there are multiple mechanisms of action for MC, and that these may be operative at different drug concentrations [2]. For example, in mice the anti-angiogenic activity of daily oral cyclophosphamide is apparent at doses of 20 mg/kg [3], yet the activation of innate anti-tumour immunity occurs on an intermittent six-day schedule and at a dose of 140 mg/kg [4]. There is some evidence that responses to low-dose daily cyclophosphamide are primarily the result of anti-angiogenic action rather than induction of anti-tumour immunity. This begs the question as to whether PK parameters can predict the outcomes of MC [5]. The key point is that low-dose drug is not a miniature version of high-dose - PK may saturate at high doses and absorption, metabolism, clearance, distribution and free drug concentration could be different. Therefore, where possible, PK sampling should be considered in clinical trials, particularly to look at correlations between plasma and intratumoral levels.

Pan Pantziarka (Anticancer Fund, UK/Belgium) briefly introduced the Repurposing Drugs in Oncology (ReDO) project, outlining the multifaceted approach that has been adopted by this collaborative project to address the issues around the re-use of non-cancer drugs in oncological treatments [6]. To date the ReDO project has published papers on mebendazole, cimetidine, clarithromycin, itraconazole, nitroglycerin and diclofenac. Future papers will include propranolol, chloroquine, disulfiram and the sartan (ARB) class of drugs. Increasingly the ReDO project is focusing on the potential of drug repurposing in the perioperative period.

Data was presented from multiple cancers suggestive of a common pattern of fast post-surgical recurrence, particularly distant recurrence. Multiple mechanisms are thought to be involved, including post-surgical immunosuppression, the release of pro-angiogenic factors as part of the wound healing response, the effects of physiological stress and psychological distress in patients [7]. A number of interventions using non-cancer drugs may be able to modulate this pattern of recurrence by acting on some of these mechanisms. The first example that was presented is ketorolac, commonly prescribed for post-operative pain. Forget *et al* had retrospectively analysed data from surgical breast cancer patients treated with different forms of analgesia and had shown that pre-surgical treatment with ketorolac was associated with a statistically significant reduction in the rate of recurrence [8]. Another example is cimetidine, a well-known antacid with a range of posited anticancer properties [9–10]. Other examples included ‘immuno-nutrition’ with l-arginine in head and neck cancers [11] and the combination of the beta-blocker propranolol and a COX-2 inhibitor [12]. Finally, the case was made for a number of possible peri-operative trials in different cancers, including a trial of pre-operative ketorolac in osteosarcoma.

### Design of metronomic studies

Aparna Parikh (PRA Health Sciences, India) outlined the use of single arm studies in metronomic therapy. The majority of these are Phase I and Phase II studies, with small patient numbers and often when standard of care has been exhausted. A meta-analysis of studies of low-dose MC (n = 80), found that the most commonly used drugs were cyclophosphamide (43%), followed by capecitabine, etoposide and vinorelbine [13]. Data from Indian studies (n = 30) include 1390 patients, with head and neck cancer patients (n = 544) and breast cancer (n = 260) being the most common. The most common metronomic therapy combination used methotrexate and celecoxib, drugs that are easily available and inexpensive.

Sudeep Gupta (Tata Memorial Centre/ACTREC, India) discussed the use of randomised Phase II studies. Phase II trials provide the testing ground for development of definitive phase III trials of new drugs, but in the case of metronomic treatment it is not a ‘new drug’ but a ‘new dose’ and ‘new schedule’. In general Phase II designs have limited sample sizes, involve compromises on type I/II errors and cannot be used to draw definitive conclusions. Key differentiators are between single and multi-arm trials, with multiple design types for each. The Gehan, Fleming and Simon trial designs were outlined. A key issue with single arm studies is the use of historical controls – and this can be problematic due to patient drift, differences in radiological technique and inter-institutional variability. For example a 2007 review of 134 phase II trials reported that 52% required historical data, of these nearly half did not cite the source of data and none incorporated statistical methods to account for sampling error, or possible differences in case mix between the sample and the historical cohort [14]. Response, in terms of tumour kill, may also be problematic in that metronomic therapy may improve OS or PFS without necessarily reducing tumour volumes. PFS has become a preferred endpoint for such phase II trials, particularly as it requires shorter follow-up and is not impacted by salvage treatments. However, when OS is generally short or salvage treatments not available, OS may be the preferred end-point. Issues with randomised Phase II designs were also discussed, with examples. In summary, a single arm trial is appropriate with good historical controls, but in many cases this is not possible and therefore a randomised Phase II design is better suited – but requires larger patient sample size and careful selection of end-points.

Kumar Prabhash (Tata Memorial Hospital, India) discussed issues related to randomised Phase III trials in metronomics. The first point is to ask the question whether Phase III trials are even necessary – in recent years there have been a number of high-profile drug approvals based on Phase II data, for example pembrolizumab for the treatment of NSCLC. However, there are many questions related to MC which are best addressed using Phase III randomised trials: looking for improvements in survival, local control or quality of life (QoL), reductions in toxicity or improvements in drug administration, improvements in patient selection, exploring treatment options for patients unfit for standard chemotherapy or for patients in which IV chemotherapy is difficult after definitive treatment. Examples of MC trials in each of these situations were outlined. Also highlighted were examples of trials where metronomic treatments had failed, for example the failure of celecoxib to show benefit in the STAMPEDE trial [15].

### Biomarkers and End-point Selection in Metronomic Trials

Atanu Bhattacharjee (Malabar Cancer Centre, India) started with the appropriate clinical end-point selection for metronomic therapies. The selection of the metronomic ‘end-point’ has considerable influence on the reliability and interpretability of clinical trials intended to evaluate the benefit-to-risk profile of an intervention. The most important characteristic in guiding the selection of the primary metronomic endpoint is that it should provide reliable evidence about whether the intervention provides *clinically* meaningful benefit. A surrogate endpoint is an outcome measure expected to reflect changes in a clinically meaningful outcome. Validating a surrogate endpoint requires providing an evidence-based justification, often from randomized controlled clinical trials. Phase I trials in MC are problematic as commonly used end-points do not apply: toxicity, maximum tolerated dose (MTD), Dose Limiting Toxicity (DLT). The concept of metronomic scheduling requires the development of appropriate intermediate surrogate markers, which can be monitored before and after therapy - particularly related to measures of angiogenesis or other relevant mechanism of action. Recent theoretical work has focused on the detection of an optimum biological dose for MC [16].

Shubhada Chiplunkar (Tata Memorial Centre/ACTREC, India) discussed tumour immunity-related biomarkers selection. It is known that many drugs used in MC have immunomodulatory activity [17]. Multiple studies have shown that immune infiltrates are prognostic in a number of cancers [18]. One study in colorectal cancer showed that the immunoscore was superior to microsatellite instability in predicting survival [19]. With multiple sub-populations of immune cells active in the tumour and microenvironment there are many assays and techniques available for analysis and selection of potential biomarkers. Data was presented showing IL17 and IFN-γ producing CD4 and CD8 T cells in oral cancer patients. In terms of immunosuppressive cell types, data was presented on myeloid-derived suppressor cells and regulatory T (T-reg) cells in oral cancer patients. The potentially pro-tumour actions of the immune system were also high-lighted, in particular the role of IL17-secreting Tγδ17 cells which can upregulate angiogenesis [20].

Sadhana Kannan (Tata Memorial Centre/ACTREC, India) outlined issues related to efficacy versus toxicity in end-point selection. The starting point is the study setting: neo-adjuvant - tumour size reduction and increase lifetime; peri-operative –reduction of metastatic rate and increase lifetime; adjuvant – increase lifetime; palliative – symptom decrease and QoL improvement. There are multiple end-points and toxicity measures appropriate to these different settings when using metronomic therapies [13]. The choice of these end-points has an impact on sample size and study design. In terms of risk benefit analyses Q-TWiST (Quality-Adjusted Time Without Symptoms and Toxicity) was highlighted as appropriate for metronomic studies [21].

## Mathematics and Biology

### Mathematics and Modelling

Sébastien Benzekry (INRIA, France) presented the use of mathematical modelling in the scheduling of chemotherapy. The topic was introduced by looking at the models which underpin the use of MTD chemotherapy – starting with a simple model of exponential growth and linear doubling time of proliferating cancer cells to the log-kill hypothesis, established in leukemic cell lines, that suggested that a given dose killed a fraction of cells [22]. However tumour growth is not constant and decelerates over time, leading to the development of the Gompertzian model and the Norton-Simon hypothesis that treatment affects the proliferative fraction of tumour cells [23]. A consequence of this was the dose densification of chemotherapy, validated in a Phase III clinical trial in breast cancer [24]. The focus remained on maximising tumour kill, however tumour heterogeneity, and the emergence of resistant clones during treatment, limited efficacy. In this context an alternative approach focused on long-term tumour minimisation emerged, with metronomic scheduling as a central mechanism. Mathematical modelling supported an anti-angiogenic mechanism of action due to the differential re-sensitisation of endothelial versus tumour cells in response to treatment [25].

Recently a dedicated model was developed for MC predicated on three hypotheses:

- Chemotherapy has an anti-angiogenic effect by killing proliferating endothelial cells
- Cancer cells develop resistance to chemotherapy, whereas endothelial cells are less likely to do so
- At low doses the cytotoxic effect of chemotherapy is greater in endothelial cells than in cancer cells

The model incorporates PK/PD parameters and evaluates the impact in terms of efficacy and toxicity [26]. An on-going clinical trial is assessing the toxicity and scheduling of metronomic oral vinorelbine in non-small cell lung cancer based on such a model (NSCLC) [27]. Mathematics has also been used to support other novel approaches, for example ‘adaptive therapy’, in which treatment is tailored to tumour response, is driven by evolutionary modelling [28]. In conclusion, although mathematical formalism may seem far removed from clinical practice, there is a need for rationally guided design of treatment protocols, both in terms of scheduling of treatment and in the sequencing of combination therapies [29].

Nicolas André (MGHI/INSERM, France) discussed the use of MC as a strategy for dealing with tumour heterogeneity. Basic evolutionary theory suggests that tumours are subject to evolutionary pressures, and it has long been known that tumours harbour clonal subpopulations [30] and that high levels of intra-tumour heterogeneity has implications in terms of response to treatment [31]. For example, Wagle *et al* reported on a case of a stunning remission of metastatic melanoma treated with the BRAF inhibitor (PLX40032), which widely relapsed after 23 weeks due to a mutation in MEK1 [32].

Recent work has sought to explore this phenomenon and the impact that chemotherapy dose, MTD versus metronomic, can have on this heterogeneity. Two NSCLC cell lines were used, A549 which is sensitive to treatment (with paclitaxel and cyclophosphamide), and EpoB40 which is resistant. 2D and 3D co-cultures, without treatment, showed that the sensitive cell line had a greater rate of proliferation and kept the resistant cell line in check compared to monoculture. Furthermore supernatant from the sensitive cell line reduced the growth rate of resistant cells – suggesting that this growth inhibition is mediated by a soluble factor. *In vitro* treatment with MTD chemotherapy caused a decrease in sensitive cell counts and a concomitant increase in the resistant cells. In the case of 3D co-cultures, MTD treatment also caused an increase in resistant cells in contrast to a decrease in non-treated spheroids. In contrast metronomic treatment limited the proliferation of both sensitive and resistant cells and limited overall growth of 2D cultures and 3D spheroids.

In conclusion, in this model the chemo-sensitive cancer clonal sub-population secreted a soluble factor that acted to inhibit the relative growth of the chemo-resistant sub-population. MTD chemotherapy reduced the number of chemo-sensitive cells, which had the effect of contributing to the growth in resistant cell numbers. In contrast, low-dose MC only partially reduced sensitive cell numbers, which were still able to repress the growth of resistant cells and therefore helped to reduce the rate of cancer growth.

### Biological Studies

Yuval Shaked (Technion, Israel) discussed the systematic analysis of host and tumour responses to MC regimens. All forms of cancer treatment, including chemotherapy, radiotherapy and surgery have effects on non-cancer cell populations. This prompts the question as to whether the tumour microenvironment is changed during therapy [33]. MTD chemotherapy can induce acute mobilization and recruitment of circulating endothelial progenitors (CEPs) to tumours and the release of pro-tumour cytokines such as G-CSF, SDF-1α and VEGF-A. Moreover, therapy may sometimes induce host effects which in turn can promote tumour cell dissemination and acceleration of metastasis. For example, it has been shown that mice ‘pre-conditioned’ with gemcitabine or paclitaxel therapy and subsequently injected with tumour cells resulted in increased mortality compared to mice pre-conditioned with vehicle control [34]. Similarly, plasma samples from colon cancer patients or mice following treatment with FOLFOX induced increased tumour cell invasion properties as assessed in vitro [35]. Data shows that radiotherapy also elicits similar effects and that locally irradiated mice intravenously injected with colon carcinoma cells succumbed to pulmonary metastasis earlier than their respective controls [36].

It has been shown that MTD and low-dose metronomic (LDM) chemotherapy have opposite effects on the mobilization and viability of CEPs [37]. Data from Robert Kerbel’s lab shows the differential host effects in response to MTD versus LDM paclitaxel in non-tumour bearing mice, with reduced expression of G-CSF and SDF-1α in response to LDM. An increase in survival had also been shown using a ‘chemo-switch’ approach with MTD treatment followed by LDM in mouse models of pancreatic, breast, prostate cancers and erythroleukemia [38–40]. The CAIRO3 study, which explored the use of bevacizumab and metronomic capecitabine as maintenance therapy following MTD treatment in treatment naïve metastatic colorectal cancer patients, recently reported improved PFS (HR = 0·67, 95% CI = 0·56–0·81, P < 0·0001) [41]. Finally, new data was presented based on mass spectrometry flow cytometry (CyTOF) as a method to systematically test host and tumour effects following therapy. The data showed changes in different stromal cell populations in tumours from mice treated with capecitabine administered at MTD or LDM dosing. To conclude, data showed that treatment elicits positive and negative (yin and yang) effects on tumour and host cells, and that these influence long-term anti-tumour responses [42]. Thus, one possible mechanism to explain the therapeutic efficacy of LDM therapy is by the minimally generated host pro-tumorigenic and pro-metastatic effects when compared to MTD therapy.

Francesco Bertolini (European Institute of Oncology, Italy) discussed MC and immunity. Early results from the teams led by Judah Folkman and Robert Kerbel showed that low-doses and frequent scheduling of chemotherapeutics such as cyclophosphamide and vinblastine showed anti-tumour efficacy despite an apparent lack of tumour cell cytotoxicity. Thus MC was initially described as an anti-angiogenic treatment, with further research showing that the mobilisation and viability of CEPs is reduced by low-dose cyclophosphamide, in contrast to the response with high-dose treatment [37]. Further studies showed that circulating peripheral blood endothelial cells (CECs) and/or their putative progenitor subset correlated with tumour neo-angiogenesis in different murine models and with response to anti-angiogenic treatments, suggesting that these could act as surrogate pharmacodynamic markers [43]. Clinical data from metastatic breast cancer patients treated with MC showed that response correlated with CEC count and viability [44]. In particular, it was shown that MC induces a release of apoptotic CECs, most likely of tumour origin. Furthermore it was shown in a panel of animal models that the maximum anti-tumour effect of metronomic treatment, with a range of chemotherapeutic drugs, correlated with maximal anti-angiogenic effect [45]. Turning to the issue of resistance or relapse after MC, analysis of breast cancer patients treated with metronomic cyclophosphamide and capecitabine with fortnightly bevacizumab showed that relapse was associated with an increase in VEGF-A and low CEC counts, possibly suggestive of a switch to an alternative form of tumour vascularisation [46]. It is suggested, therefore, that combinations of MC with anti-VEGF treatments are used in association with drug repurposing to block multiple forms of vascularisation [47].

More recently there has been a focus on the immune effects of chemotherapeutic drugs [48] – with suggestions that MC has immunological activity in addition to anti-angiogenic activity [49]. In particular there is an interest in encouraging immunogenic cell death in the context of checkpoint inhibition [50]. In terms of repurposed drugs, the anti-diabetic drug metformin has been an object of considerable research interest, with retrospective analyses showing a survival benefit in diabetic cancer patients [51]. Among the possible anti-cancer mechanisms there is evidence for an anti-angiogenic effect [52], as well as induction of apoptosis in breast cancer cells [53]. Preliminary data was presented that suggested that metformin treatment reduces certain populations of immunosuppressive cells and may have antagonist effects when used following checkpoint inhibitor treatment. In contrast, as yet unpublished data suggests that MC may synergise with checkpoint inhibition, thus opening the door to new combination therapies.

Mark Kieran (Dana-Farber Cancer Institute, USA) discussed the role of resolvins in the regulation of the tumour microenvironment. MC could well be characterised as microenvironment therapy, with effects on both endothelial cells and on inflammatory/immune cells. The traditional view held that cancer was a cell-autonomous disease, later, under the influence of Judah Folkman, it became viewed as a disease of cancer cells and supporting endothelial cells. Now, it is increasingly viewed as a tissue or developmental disease that includes cancer, endothelial, stromal, inflammatory/immune and circulating progenitor cells. Serhan *et al* have outlined the role of resolvins, a family of lipid mediators, in the resolution of the inflammatory response to injury or microbial infection [54–55]. In particular, incomplete phagocytosis results in the failure of the acute inflammatory response to resolve, thereby leading to a chronic inflammatory response. Resolvins act to both enhance macrophage phagocytosis and efferocytosis, and also have anti-inflammatory effects via down-regulation of neutrophil recruitment, IL-1, IL-6 and TNF-α [56]. Laszlo Revesz had noted, in 1956, that implanting a mixture of viable and non-viable (x-ray treated) cancer cells in mice resulted in an increased rate of tumour growth and reduced survival time compared to implanting viable cancer cells alone [57].

The hypothesis was presented that therapy-induced cell death and incomplete efferocytosis may therefore lead to increased tumour growth, and that improving efferocytosis or resolution of inflammation may inhibit that growth. Laboratory evidence showed that cancer cells injected at sub-threshold inoculum result in tumour dormancy [58], and that the addition of tumour cell debris (apoptotic and necrotic cells) leads to escape from dormancy and primary tumour growth. Furthermore recent studies show that resolvins are able to inhibit this apoptosis-driven tumour growth. Results suggest that resolvins act on macrophages to induce an ‘M-Res’ phenotype with increased phagocytic activity and therefore inhibit apoptosis-driven tumour growth. These results suggest therapeutic enhancement of endogenous resolution as a novel modality to complement current cancer treatments that inevitably generate tumour cell debris.

David Waxman (Boston University, USA) discussed MC-stimulated anti-tumour immunity. The traditional view has been that MC is primarily anti-angiogenic in nature, with endothelial cells as the main targets. However, increasingly MC has been shown additionally to have anti-tumour activity against infrequently cycling tumour cell sub-populations and to have effects on the innate and adaptive immune system. Metronomic cyclophosphamide, on a six-day schedule, has been shown to regress large, established glioma xenografts. If the main mechanism of action were anti-angiogenic then we would expect synergy with anti-VEGF treatments, however, combination with the VEGFR inhibitor axitinib abolishes the anti-tumour effect of metronomic cyclophosphamide without impacting the anti-angiogenic effect [59]. Subsequent work demonstrated that the six-day schedule of metronomic cyclophosphamide induces recruitment of cells from the innate immune system (macrophages, dendritic and NK cells), and that this recruitment was blocked by anti-VEGF treatment, thereby blunting the anti-tumour effect [60]. In part this is because metronomic cyclophosphamide induces immune cell infiltration and activation of the NK cell cytotoxic effectors perforin and Gzmb, all of which are blocked by VEGF/VEGFR signalling inhibitors. This block in immune stimulation is associated with VEGFR inhibition, but not with anti-angiogenesis *per se*, as shown using the anti-angiogenic agent sorafenib under conditions where it strongly inhibits angiogenesis by a VEGFR-independent mechanism and does not block the immunogenic effects of metronomic cyclophosphamide [61].

The immune stimulatory effect of metronomic cyclophosphamide is time and dose dependent, and there is evidence that the six-day schedule is optimal in the immune-mediated ablation of implanted murine glioblastomas, which in fully immune-competent mice is associated with activation of cytotoxic CD8-T cells and long-term, tumour-specific immune memory [62]. Extending the gap between treatments to more than six days downregulates the NK and CD8-T cell responses, and also upregulates an immunosuppressive T-reg response. Intermittent treatment is essential to induce an innate anti-tumour immune response [4], in contrast to the low-dose daily dosing most often used in clinical MC regimens with cyclophosphamide. Recent work indicates that six-day metronomic cyclophosphamide treatment of glioma xenografts activates the interferon signalling network [63], and that tumours showing metronomic cyclophosphamide-induced immune responses have high basal levels of immune infiltration compared to tumours that are intrinsically sensitive to cyclophosphamide cytotoxicity, but do not respond to metronomic cyclophosphamide with a robust immune response [64]. More research is required to: ascertain whether or not the tumour-cell-based interferon responses are both necessary and sufficient for tumour regression; to identify other drugs and tumours that show this schedule-dependent immune response; and finally, to identify gene signatures to predict patient responsiveness to this immune-stimulatory metronomic schedule.

## Plenary Talks/Keynote Lectures

Eddy Pasquier (MGHI/INSERM, France) introduced the concept of metronomics, which is defined as the combination of drug repurposing and MC [65], using the example of the non-selective beta-blocker propranolol. The serendipitous observation that propranolol was an effective and non-toxic treatment of severe infantile hemangioma [66] raised the question as to whether similar anti-angiogenic effects might be active in vascular tumours. A number of epidemiological studies were suggestive of a positive impact of beta-blockers on cancer outcomes [67–68]. Pasquier and André hypothesised that propranolol might be active in combination with chemotherapy and their pre-clinical work indeed showed that propranolol was able to potentiate the anti-tumour effects of chemotherapy in both breast cancer and neuroblastoma models [69–70]. This work was extended to angiosarcoma, a rare soft-tissue cancer with poor prognosis when metastatic. A ‘bench to bedside’ approach was adopted in which it was shown that propranolol and vinblastine had synergistic activity against angiosarcoma cell lines *in vitro* and clinical use of vinblastine-based MC in combination with propranolol produced consistent and durable responses in a small number of patients [71–72].

The traditional drug development process starts with identification of novel therapeutic targets prompting high-throughput screening of libraries to identify pharmacological ‘hits’ and ultimately leads to the development of novel therapeutics and drug approval – a process that can take 17–23 years from start to finish. In contrast, a next generation drug repurposing model was outlined – starting with screens of approved drugs based on the principle of ‘synthetic lethality’, identification of active molecules and their targets, validation of key genes by siRNA screening and then the development of new treatment modalities and validation of associated biomarkers (*in vivo* testing, predictive/prognostic biomarkers and functional validation). An example of this approach was presented, focusing on glioblastoma multiforme, a disease with a dismal prognosis. Starting with a screen of around 3700 approved drugs and pharmacologically active molecules, alone and in combination with temozolomide or erlotinib around 80 ‘GBM killers’ and 40 ‘treatment enhancers’ were identified – including many approved drugs from multiple families (e.g. chemotherapeutics, anti-helminthics, antibiotics, anti-psychotics etc). Bioinformatic analysis revealed over 1200 known molecular targets for these pharmacological hits and high-throughput siRNA screening was used, again based on synthetic lethality, to filter down the most promising targets. This work is now pending *in vivo* validation but could lead to the discovery of approved drugs that could synergise with standard of care drugs used in GBM, together with their associated biomarkers to be used for patient selection in the clinic.

Robert Kerbel (Sunnybrook Research Institute, University of Toronto, Canada) discussed a reassessment of the anti-tumour mechanisms of MC. Initially, based on the work of Judah Folkman’s and Kerbel’s groups, MC was characterised as primarily anti-angiogenic [73–75]. However, the positive results from both animal models and small patient trials, in addition to further elucidation of the anti-vascular role of MC have yet to lead to its widespread adoption. The advantages, including reduced acute toxicity, increased patient convenience through the use of oral drugs, practicality of long-term adjuvant or maintenance use and the ease with which metronomic treatments can be combined with targeted or other therapies [49] have been insufficient to overcome a number of negative perceptions. Chief among these are the fact that the precise mechanisms of action are still rather vague and a question mark about the clinical relevance of the animal data (which largely used ectopic subcutaneous and transplanted primary tumours).

A response to this has been the use of more relevant animal models, particularly the use of mouse models of spontaneous metastases [76]. An illustrative example of the difference between primary tumour responses to treatment compared to the response of metastatic diseases is shown by the experience of sunitinib. While sunitinib, which failed to show efficacy in four large Phase III trials in metastatic disease, was shown to be effective against primary tumours it failed to show to show an effect in a model of post-surgical metastatic disease [77]. The mechanism associated with this apparent resistance to anti-angiogenic treatments in metastatic disease may be related to vessel co-option [78]. In some tissues, particularly in the liver and lungs, tumours are able to hijack existing vasculature thus bypassing the need for the sprouting angiogenesis which is the primary target of anti-angiogenic treatment such as sorafenib [79]. The question posed, therefore, is can the co-opted vasculature be targeted by an anti-vascular, as opposed to anti-angiogenic, therapy?

The other mechanisms of action associated with MC, particularly the role of immunity and direct cancer cell targeting were also discussed. For example the effects of low-dose metronomic cyclophosphamide on T-reg function are well-known. There is potential to combine MTD and MC (the ‘chemo-switch’ strategy) in order to prime the response which immune checkpoint inhibitors amplify. Looking to the future, Kerbel posited a multi-targeting approach that utilised MC, in combination with other treatments, to target angiogenesis, co-opted vasculature, immune stimulation and direct effects on cancer cells.

Dan Duda (Massachusetts General Hospital, USA) discussed tumour vascular normalisation as a paradigm shift for cancer treatment with anti-angiogenics. The starting point is to seek answers to the key challenge in anti-angiogenic therapy: the inability to control tumour growth in patients with anti-angiogenics. Noting that there are multiple mechanisms by which tumours are able to acquire new blood vessels [80], the question is whether anti-angiogenic therapy is failing due to the lack of efficient anti-vascular effects. The failures of anti-angiogenic therapy in both highly vascularized (glioblastoma, hepatocellular carcinoma) and poorly vascularized (pancreatic adenocarcinoma) cancers indicate that the anti-vascular (vessel pruning) effect is unlikely to be a key mechanism of benefit. Moreover, anti-angiogenics have consistently shown efficacy when combined with chemotherapy in lung and colorectal cancers. The paradox is that anti-angiogenics are designed to destroy the tumour vasculature, which is necessary for delivery of concomitantly administered cytotoxics. The vascular normalisation hypothesis proposes that by normalising the tumour vasculature chemotherapy and radiotherapy can become more effective and therefore confer a survival benefit [81]. Additionally, vascular normalisation can reduce hypoxia, reverse low pH in the tumour microenvironment and decrease the high interstitial pressure. Data from non-small cell lung cancer (NSCLC) patients treated with bevacizumab and chemotherapy showed that improved perfusion was associated with superior OS after chemotherapy [82]. Data from breast cancer patients similarly showed that benefit was associated with normalisation of vasculature, and that pre-treatment vessel density was a possible indicator of potential benefit [83]. In terms of MC, there are some indications that vascular normalisation may be a relevant mechanism of action.

Finding biomarkers that associate with benefit is another unmet need for the use of anti-angiogenic therapy for cancer [84–85]. A number have been proposed, including a ‘vascular normalisation index’ that correlates with survival in recurrent glioblastoma [86], plasma soluble VEGFR1 [87] and others, all of which need to be prospectively validated.

Finally, attention turned to the problem of resistance to anti-angiogenic treatments. Adjuvant and neo-adjuvant anti-VEGF therapy has not been efficacious in reducing pulmonary or lymph node metastases in colorectal and breast cancer patients. In the case of recurrent disease the evidence points to a lack of ‘rebound’ angiogenesis and an increase in inflammation and invasion as driving forces [88]. Multiple biomarkers of escape have been postulated, including Ang2, SDF1α and CXCR4. Data from an animal model of glioblastoma has shown that dual inhibition of Ang2 and VEGF leads to tumour normalisation and prolonged survival [89]. SDF1α is associated with rapid tumour progression and resistance to anti-VEGFR therapy in HCC [90]. Therefore inhibition of SDF1α may be a viable option to explore clinically [91–92]. In doing so there is also a potential for synergistic interaction with anti-PD1 checkpoint inhibitors [93].

## Symposia

### Symposium on breast cancer

Antonella Palazzo (European Institute of Oncology, Italy) discussed the Italian experience of MC in breast cancer. Noting the increase in the number of papers published on MC in breast cancer [94], Palazzo briefly outlined the range of clinical studies, including combination metronomic, single agent metronomic, combination with hormonal therapies and combinations with biological agents before moving to a number of recent trials in different disease settings. In the neo-adjuvant setting a Phase II trial of epirubicin, cisplatin, and infusional fluorouracil followed by weekly paclitaxel with metronomic cyclophosphamide was used as a preoperative treatment of triple-negative breast cancer (TNBC) (n = 34) [95]. The primary outcome was a reduction in Ki67 staining pre and post-treatment, with clinical response as secondary outcome. The results showed a reduction of Ki67 of 41% (95% CI = 30%-51%; P < .0001), and a pathological complete response rate of 56% (95% CI = 35% -70%). These results are improved compared to previous studies but without an increase of major adverse events.

In the adjuvant setting Colleoni *et al* reported at ASCO 2015 on the IBCSG 22-00 Phase III randomised controlled trial of metronomic cyclophosphamide-methotrexate maintenance (CMM) for receptor-negative early breast cancer (BC) [96]. Patients (n = 1086) were assigned to CMM (542) or observation (539) following adjuvant chemotherapy. Results showed a 5-year DFS of 78.1% for CMM versus 74.7%, HR = 0.84 (95% CI = 0.66 - 1.06, P = 0.14). However, for node positive, triple negative patients the degree of benefit was greater, with HR = 0.71 (95% CI = 0.49 - 1.03). A total of 64 patients, 13.5% of those receiving at least one dose of CMM, had a grade 3 or 4 treatment-related adverse event (AE), there were no grade 5 AEs reported. Additionally, a translational study analysed 672 patient samples for tumour-infiltrating lymphocytes (TILs) [97]. The median TILs score was 18%, and 119 patients had TILs ≥ 50%, defining a lymphocyte-predominant breast cancer (LPBC) phenotype. When assessed as a binary variable (LPBC vs. non-LPBC), the estimated HR for breast cancer-free interval was 0.56 (95%CI = 0.33-0.94, P = 0.03) in univariate analysis. These results suggest that higher TILs may be predictive of clinical benefit from CMM therapy. Finally, in the metastatic setting the VEX trial (metronomic cyclophosphamide, capecitabine and vinorelbine) is a Phase II study to test the tolerability and efficacy of the three drug oral combination in women with hormone receptor positive metastatic disease [98]. Preliminary data presented at the meeting showed that of 88 evaluable patients, n = 46 were treatment-naïve and n = 42 were pre-treated. In the treatment-naïve patients the response rate (RR) was 35%, with median time to progression (TTP) of 26.5 months and 2-year PFS of 53%. In the pre-treated group the RR was 30%, median TTP 9.6 months and 2-year PFS 28%. The treatment was well-tolerated by all patients with no grade 4 AEs reported.

Shripad Banavali (Tata Memorial Hospital, India) outlined the Indian experience of MC in breast cancer, starting with a case from 2008 of metastatic and recurrent disease showing long-term response to oral MC doublet of capecitabine and cyclophosphamide. This ultimately informed the METRO-ABC trial (NCT02664103), an Indian multi-site study of a fixed dose of capecitabine and cyclophosphamide in a single pill (SAR439281) in women with advanced breast cancer. In this open-label randomised trial the primary objectives are safety and PK/bioavailability after 12 weeks of treatment. Responding patients will be treated and followed-up until disease progression.

TNBC, with a higher prevalence in LMIC, presents with more aggressive disease and in a younger age group than other breast cancer subtypes. In India five-year OS is 37.5% in patients treated with MTD standard of care [99], but TNBC has high unmet needs globally [100]. In high-income countries new agents under investigation include PARP inhibitors, microtubule disrupters, antiangiogenic and checkpoint inhibitors, but these agents are expensive and not affordable for patients in LMIC. Therefore a low-cost metronomic maintenance (MMT) protocol has been developed for TNBC consisting of daily celecoxib (200 mg BID), cyclophosphamide (50 mg, daily), and IV cisplatin (25 mg/m^2^, weekly), for three months. This is followed by one year of maintenance of metformin (500 mg, BID), cyclophosphamide (50 mg, daily) and weekly methotrexate (12 mg/m^2^). MMT was used at a rural cancer centre on all newly diagnosed TNBC to prevent relapses following MTD treatment. A retrospective study (n = 48) compared MMT patients to an observation group and reported a DFS of 89.2% for MMT compared to 54.5% for the observation group [101]. Subsequently an 18-month protocol of metformin, cyclophosphamide and methotrexate is being tested at Kokilaben Hospital in Mumbai.

### Symposium on head and neck cancers

Amit Joshi (Tata Memorial Hospital, India) discussed the use of MC in adjuvant and neo-adjuvant settings in head and neck cancer. HNSCC is the third most common cancer in India, and the second commonest cancer in Indian males. The majority of patients present in an advanced stage. Long term survival (3-5 years) is 10-30% in locally advanced disease. Efforts to improve OS have been largely disappointing, but encouraging results using MC have been reported in a palliative setting [102], and other results show an improvement in two-year DFS associated with metronomic treatment [103]. On-going trials include a comparison of standard treatment (surgical resection + radiotherapy) vs oral MC in operable AJCC Stage III and IV oral squamous cell carcinoma. Another randomized study will evaluate metronomic adjuvant chemotherapy in recurrent head and neck cancers post R0 salvage surgical resection ineligible for re-irradiation. A non-randomised Phase II study in Taiwan (NCT00855881) will explore the effect of one-year treatment of metronomic tegafur-uracil in HNSCC. Proposed studies include the evaluation of metronomic adjuvant chemotherapy in locally advanced oral cancers post-surgery and appropriate adjuvant therapy, and a study of metronomic adjuvant chemotherapy in locally advanced oropharynx , larynx and hypopharynx post-definitive chemo-radiotherapy.

Vijay Patil (Tata Memorial Hospital, India) discussed MC versus MTD chemotherapy in a palliative care setting. The standard of care palliative treatment in head and neck cancers is the combination of cisplatin, 5FU and cetuximab in high income countries [104]. However, cetuximab is an expensive drug that is not affordable by the vast majority of patients in India and other LMIC, therefore standard treatment is cisplatin in this setting. It is in this context that metronomic alternatives have been sought – using in vitro and in vivo evidence to show that the combination of low dose methotrexate and celecoxib may have efficacy, followed by encouraging signs of activity in late-stage cancer patients [105–106]. A Phase II randomised trial compared single agent cisplatin to the combination of daily celecoxib and low-dose weekly methotrexate showed that OS was significantly (P = 0.02) higher in the metronomic arm (median 249 days, 95% CI: 222.5–275.5 days) compared to the cisplatin arm (median 152 days, 95% CI: 104.2–199.8 days) [102]. However, these positive results are inferior to the results with cetuximab. On-going studies are seeking to confirm efficacy compared to cisplatin and also to identify optimal doses and causes of resistance. For example a Phase I/II study of oral metronomic methotrexate with celecoxib and erlotinib as palliative chemotherapy in early failures and platinum-resistant oral cancers. Initial results in a cohort of patients (n = 15), showed some evidence of an improved PFS [107]. In terms of defining the optimal dose of metronomic methotrexate a de-escalation strategy will be adopted to ascertain whether a lower dose than 15 mg/m^2^ weekly is able to show an improved two-month clinical benefit rate. Additional translational studies are seeking to improve understanding of the mechanism of action, particularly with respect to angiogenesis.

### Symposium on pediatric oncology

Nicolas André discussed the SFCE (French Society for the Combat of Cancer and Leukemias in Children and Adolescents) experience of metronomics in pediatric oncology. Three trials were highlighted. Fluvabrex (NCT02115074) is a Phase I trial of metronomic fluvastatin and celecoxib in pilocytic astrocytoma of the optic nerve. Pre-clinical evidence suggested that the combination of these drugs was effective in cell lines, and a case report was published showing the successful treatment of a child with a refractory multifocal pilocytic astrocytoma for 18 months [108]. Ten patients have been recruited to date, with seven having stopped treatment (6 due to progression, one due to toxicity), three are continuing treatment. One more patient is required to establish level 2 dosing.

Metro-SFCE01 was a pilot trial of a four-drug regimen for the treatment of refractory or relapsing solid tumours. Weekly vinblastine, daily low dose oral cyclophosphamide, daily celecoxib and twice-weekly methotrexate [109]. In all sixteen patients were treated, and treatment was associated with low toxicity and disease stabilisation. An open label Phase II trial, NCT01285817, is now on-going and it includes six cohorts (ependymoma, neuroblastoma, bone sarcoma, soft-tissue sarcoma, miscellaneous non-brain tumours and miscellaneous brain tumours). To date the most consistent responses have been in low-grade gliomas.

Metro-SFCE02 is a Phase I/II trial that combines vinorelbine with the beta-blocker propranolol. Pre-clinical evidence shows that propranolol can potentiate the effect of microtubule disrupting chemotherapy in vitro and increased survival time in a murine model of neuroblastoma [70].

The Phase II portion of this trial will recruit four cohorts of nine patients with neuroblastoma, Ewing’s sarcoma, rhabdomyosarcoma and miscellaneous tumours.

Jaroslav Sterba (University Hospital Brno, Czech Republic) discussed theranostics and metronomic approaches to relapsed and refractory pediatric solid tumours. The focus was on experiences in the post-COMBAT [110] era, incorporating data from index cases treated in the previous two years. The context is that survival gains in pediatric oncology have plateaued since the 1990s, and that incremental gains are largely due to refinements of existing treatments rather than the development of new agents [111]. One reason may be that nearly all anti-cancer drugs used routinely in paediatrics are targeted at tumour cell DNA. The aim remains on inflicting maximum tumour cell kill – with corresponding high rates of toxicity and adverse events. Treatment of relapsed or recurrent disease remains challenging and survival after relapse remains poor. This may be related to the fact that relapsed tumours are often genetically distinct from primaries – with implications for targeted therapies [112].

Data from pediatric patients treated with a metronomics and/or theranostics approach at Brno was presented (n = 43, 34 were children with relapsed or recurrent disease and nine had high risk disease, e.g. DIPG or metastatic rhabdomyosarcoma). Results showed that time to second progression was not shorter than time to first progression for treatment with MC. Data on a number of index cases was also presented, showing some value in an individualised approach to treatment based on mechanism of action of selected agents, but the small number of patients means that conclusions are premature at this stage.

However, patients are dying and there is a need to make progress now – existing incentives mean that research remains focused on adult cancers. Sterba characterised an approach that is ‘disease-centric’ or ‘patient-centric’ to contrast with the ‘drug-centric’ approach that predominates. This approach focuses on the analysis of individual patient disease and identifying which drugs to use based on a mechanism of action of the drug. In the case of a disease like osteosarcoma, (in which each case may harbour unique molecular diversity), the theranostic approach is compelling [113]. Ultimately this requires a new paradigm in oncology, and one that exposes a tension between physicians and patients on the one hand, and regulators on the other [114].

Mark Kieran discussed a Phase II trial of a 5-drug oral anti-angiogenic regimen in children with recurrent or progressive tumours (the MEMMAT trial, NCT01356290). Patients with relapsed medulloblastoma have a very poor prognosis despite a range of treatment modalities available. This study will evaluate the use of biweekly IV bevacizumab in combination with five oral drugs (thalidomide, celecoxib, fenofibrate, and alternating cycles of daily low-dose oral etoposide and cyclophosphamide), augmented with alternating courses of intrathecal etoposide and liposomal cytarabine. The primary objective of the MEMMAT trial was to evaluate the efficacy of this multidrug anti-angiogenic approach in this heavily pre-treated patient population [115]. Additionally, safety and tolerability data was also further assessed.

Giselle Saulnier Sholler (Helen DeVos Children’s Hospital, USA) discussed the results of two clinical trials using the repurposed drug DFMO in high-risk neuroblastoma. High-risk neuroblastoma comprises around 44% of total incidence. The standard treatment in the US consists of 5-6 cycles of chemotherapy (cyclophosphamide, topotecan, cisplatin, etoposide, doxorubicin, vincristine) and surgical resection of the tumour. Consolidation treatment of autologous bone marrow transplant and radiation therapy to primary site follows. Maintenance is isotretinoin and chimeric anti-GD2 antibody. Despite this, overall survival in the high-risk group remains around 50%. Preclinical evidence showed that DFMO, a generic drug used to treat African sleeping sickness, acts as an ODC inhibitor and that ODC is over-expressed in neuroblastoma [116]. A Phase I trial of DFMO in relapsed or refractory neuroblastoma showed that the treatment was safe and tolerable (MTD not reached) [117]. While not powered for efficacy, results were positive: PFS mean 314 days / median 82 days, and with three (of 18) long-term survivors (3 -6 years).

The Phase I trial was followed by a Phase II trial of DFMO as a preventative in patients with high-risk neuroblastoma in remission (NCT01586260). There are two strata of patients – those who were successful at the end of upfront standard of care, and those who had been in relapse or suffering from recurrent disease who achieved remission. Interim analysis has shown a markedly superior EFS and OS compared to historical controls. Accounting for all potential forms of bias the differences in EFS and OS outcome in these cohorts are greater than can be accounted for by any of the potential sources of bias. Survival data for both strata of patient suggest that metronomic DFMO in all groups, including patients with relapsed or refractory disease is improved. Publication of two-year survival results are planned by the end of 2016.

Jaroslav Sterba discussed the metronomic principles behind the I-BFM LL09 protocol for lymphoblastic lymphomas.

## Other

### Best paper presentations

Gautam Goyal discussed a Phase II study of oral MC in relapsed epithelial ovarian cancer [118]. Hitesh Khatwani reported on oral MC in metastatic triple negative breast cancer [119]. Vanita Noronha discussed a prospective randomised Phase II study comparing MC versus single agent cisplatin in patients with metastatic, relapsed or inoperable squamous cell carcinoma of the head and neck [102]. Avinash Pandey discussed a retrospective study of outcomes in operable oral cavity cancer in rural India [120].

### Case Reports

Jyoti Bajpai and Deepa Philip outlined a number of interesting cases, including angiosarcoma and geriatric cancers. Vikas Ostwal and Bhausaheb Bagal outlined interesting cases in GI cancer and lymphoma respectively.

### 2^nd^ Barton Kamen/MGHI Prize

This was awarded to Giselle Saulnier Sholler for her pioneering work on the use of the repurposed drug DFMO in high-risk neuroblastoma.

### Other

Mubarak Naqvi of Sanofi discussed a project developing a fixed-dose combination pill for use in metronomic therapy. Abishek Mahajan and Nilendu Purandare outlined issues related to response assessment in metronomic therapy, looking at the radiological and nuclear medicine view-points respectively.

Vijay Patil delivered the valedictory speech at the end of an interesting and successful meeting.

## Concluding Remarks

Some of the themes of this meeting are similar to those reported in the previous meeting in Milan in 2014 [121]. There remains a good deal of active research to further elucidate the mechanisms of action of MC, particularly with respect to anti-angiogenesis, the immune response and direct effects on cancer cells. There is an acknowledged need to identify biomarkers predictive of response to MC and to address mechanisms of resistance. Despite continuing progress with clinical trials, MC still suffers from not being seen as a mainstream treatment option except in the case of maintenance or palliative settings. Indeed, in some cases the words metronomic are not even used when clearly the dose and the schedule of a chemotherapeutic drug clearly fits in with the generally accepted definition [122].

And yet in spite of these recurring concerns the field remains innovative and forward looking. The range of clinical trials is increasing, encompassing all disease settings, not just the palliative. There is a greater interest in the possibilities of combining MC with repurposed non-cancer drugs. And there is a renewed interest in the combination with targeted therapies and immunotherapeutics.

Finally, while both metronomics and drug repurposing suffer from problems of financial disincentives in high income economies the low costs associated with these treatments are a positive in low and middle income countries. It was therefore a highlight of this meeting to hear at first hand reports of innovative and credible clinical results achieved in India using these approaches. Indeed it is to be hoped that with continued positive results we may begin to see clinicians in high income countries learn from these experiences.

## Author Contributions

Primary author: Pan Pantziarka. All authors read and approved the final manuscript.
